# Supplementary material for: The Petunia CHANEL Gene is a ZEITLUPE Ortholog Coordinating Growth and Scent Profiles
Source: Cells. 2019 Apr 11;8(4):343. doi: 10.3390/cells8040343 (PMC6523265; doi:10.3390/cells8040343)
Supplement: Supplementary file 1 [file cells-08-00343-s001.zip › Supplemental Table S1 Accessions.docx]

**Table S1**. GenBank, Snapdragon Genome Sol Genomics Network Database accession numbers of proteins used in phylogenetic reconstruction.

| **Species** | **Protein** | **Accession** | **Database** |
| --- | --- | --- | --- |
| *Antirrhinum majus* | ZTL | Am01g33620 | Snapdragon |
| *Arabidopsis thaliana* | FKF1 | OAP14729.1 | GenBank |
|  | LKP2 | NP_849983.1 | GenBank |
|  | ZTL | OAO90691.1 | GenBank |
| *Cryptomeria japonica* | ZTL | BAP76060.1 | GenBank |
| *Hordeum vulgare* | ZTL | ACR15149.1 | GenBank |
| *Marchantia polymorpha* | FKF | BAO66508.1 | GenBank |
| *Mesembryanthemum crystallinum* | ZTL | AAQ73527.1 | GenBank |
| *Nicotiana attenuata* | ZTL | AFA35963.1 | GenBank |
| *Oryza sativa* | ZTL | XP_015643178.1 | GenBank |
| *Panicum hallii* | ZTL | XP_025810803.1 | GenBank |
| *Petunia axillaris* | CHL | Peaxi162Scf01124g00126.1 | Sol Genomics |
|  | FKF | Peaxi162Scf00655g00114.1 | Sol Genomics |
| *Petunia inflata* | CHL | Peinf101Scf01230g02037.1 | Sol Genomics |
|  | FKF1 | Peinf101Scf04186g00007.1 | Sol Genomics |
|  | FKF2 | Peinf101Scf02808g00015.1 | Sol Genomics |
| *Picea abies* | ZTL | AGH20050.1 | GenBank |
| *Solanum lycopersicum* | FKF | Solyc01g005300.3 | Sol Genomics |
| *Triticum aestivum* | ZTL | ABR14627.1 | GenBank |
| *Zea mays* | ZTL | PWZ17109.1 | GenBank |
