# Supplementary material for: The Petunia CHANEL Gene is a ZEITLUPE Ortholog Coordinating Growth and Scent Profiles
Source: Cells. 2019 Apr 11;8(4):343. doi: 10.3390/cells8040343 (PMC6523265; doi:10.3390/cells8040343)
Supplement: Supplementary file 1 [file cells-08-00343-s001.zip › Supplemental Table S2 Primer list.docx]

**Table S2**. Primers for PCR (attb1, attb2, PhCHLrnaiaattb1, PhCHLrnaiaattb2, Agri51, Agri 56, NPTII and specific fragment of *PhCHL* for transformation, *RNAi:PhCHL*) and qPCR (*PhACT*, *PhCYP*, *PhEF1α*, *PhGADPH*, *PhFKF*, *PhRAN1*, *PhRPS13*, *PhUBQ* and *PhCHL.*

| Gene | Forward | Reverse |
| --- | --- | --- |
| attb1, attb2 | GGGGACAAGTTTGTACAAAAAAGCAGGCT | GGGGACCACTTTGTACAAGAAAGCTGGGTA |
| PhCHLrnaiaattb1 | *TACAAAAAAGCAGGCTCT*GCATGAACTATCTTTAGCAAGC |  |
| PhCHLrnaiaattb2 | *CAAGAAAGCTGGG*TAATTCAGACCATCTGCTCAATAGG |  |
| Agri51,Agri56 | CAACCACGTCTTCAAAGCAA | CTGGGGTACCGAATTCCTC |
| NPTII | CCTGCTTGCCGAATATCATGGTGG | CGAAATCTCGTGATGGCAGGTTGG |
| *RNAi:PhCHL* | TGCATCTGTTGGCTCTGTTT | CCCCAACCCAATCTCTTAGC |
| *PhACT* | TGCACTCCCACATGCTATCCT | TCAGCCGAAGTGGTGAAAGAG |
| *PhCHL* | TGCATCTGTTGGCTCTGTTT | CCCCAACCCAATCTCTTAGC |
| *PhCYP* | AGGCTCATCATTCCACCGTGT | TCATCTGCGAACTTAGCACCG |
| *PhEF1α* | CCTGGTCAAATTGGAAACGG | CAGATCGCCTGTCAATCTTGG |
| *PhGADPH* | AACAACTCACTCCTACACCGG | GGTAGCACTAGAGACACAGCCTT |
| *PhFKF* | CTGGGCAACCTCCAAAGTT | CATGGATCAGAATCTTGTT |
| *PhRAN1* | AAGCTCCCACCTGTCTGGAAA | TGCTAACAGATTGCCGGAAGCC |
| *PhRPS13* | CAGGCAGGTTAAGGCAAAGC | ACTAGCAAGGTACAGAAACGGC |
| *PhUBQ* | TGGAGGATGGAAGGACTTTGG | CAGGACGACAACAAGCAACAG |
