# Supplementary material for: The Petunia CHANEL Gene is a ZEITLUPE Ortholog Coordinating Growth and Scent Profiles
Source: Cells. 2019 Apr 11;8(4):343. doi: 10.3390/cells8040343 (PMC6523265; doi:10.3390/cells8040343)
Supplement: Supplementary file 1 [file cells-08-00343-s001.zip › Supplemental Table S3 BLAST.docx]

**Table S3.** Results from BLAST of the *Petunia* genomes and major features of homology of the CHL/FKF/ZTL protein family. Comparisons are made against the Arabidopsis ortholog. The homology refers to the number of identical annealed amino acids in relation to the total coded protein.

| **Petunia gene** | **BLAST values** | **Predicted protein length** | **Homology to Arabidopsis** |
| --- | --- | --- | --- |
| PhCHL Peaxi162Scf01124g00126.1 | 0.0 | 616 | 489/610 |
| PhFKF Peaxi162Scf00655g00114.1 | 0.0 | 617 | 478/582 |
| PinfZTL Peinf101Scf01230g02037.1 | 0.0 | 623 | 502/610 |
| PinfFKF1 Peinf101Scf04186g00007.1 | 0.0 | 593 | 479/582 |
| PinfFKF2 Peinf101Scf02808g00015.1 | 0.0 | 618 | 461/577 |
