# Supplementary material for: The Petunia CHANEL Gene is a ZEITLUPE Ortholog Coordinating Growth and Scent Profiles
Source: Cells. 2019 Apr 11;8(4):343. doi: 10.3390/cells8040343 (PMC6523265; doi:10.3390/cells8040343)
Supplement: Supplementary file 1 [file cells-08-00343-s001.zip › Supplemental Table S4 Analysis of leaf mov.docx]

**Table S4**. Analysis of leaf movement in *RNAi:PhCHL* and non-transgenic segregating siblings (PhWT). Images were analyzed using the JTK algorithm in the Metacycle package. Significant rhythms were tested using the Bejamini-Hochberg critical value (BH.Q). Adjusted P-value (ADJ.P) was used as a cut-off to identify clock-regulated transcripts. When both of them are less than 0.05, the circadian rhythm is accepted. Period is the time lapse between to peaks. LAG is the time of the first peak. Amplitude is the distance between the average of an oscillation and the peak.

| **Group** | **Tissue** | **BH.Q** | **ADJ.P** | **Period (h)** | **LAG (phase)** | **Amplitude** |
| --- | --- | --- | --- | --- | --- | --- |
| PhWT | Leaf 1 | 6.38e^-14^ | 3.19e^-14^ | 25 | 11 | 0.1160 |
|  | Leaf 2 | 4.30e^-13^ | 4.30e^-13^ | 24 | 12.5 | 0.2106 |
| RNAi:CHL | Leaf 1 | 9.663e^-05^ | 9.66e^-05^ | 25 | 12.5 | 0.1615 |
|  | Leaf 2 | 1.64e^-14^ | 8.21e^-05^ | 24 | 11.5 | 0.1283 |
