# Supplementary figures and images for: The Petunia CHANEL Gene is a ZEITLUPE Ortholog Coordinating Growth and Scent Profiles
Source: Cells. 2019 Apr 11;8(4):343. doi: 10.3390/cells8040343 (PMC6523265; doi:10.3390/cells8040343)

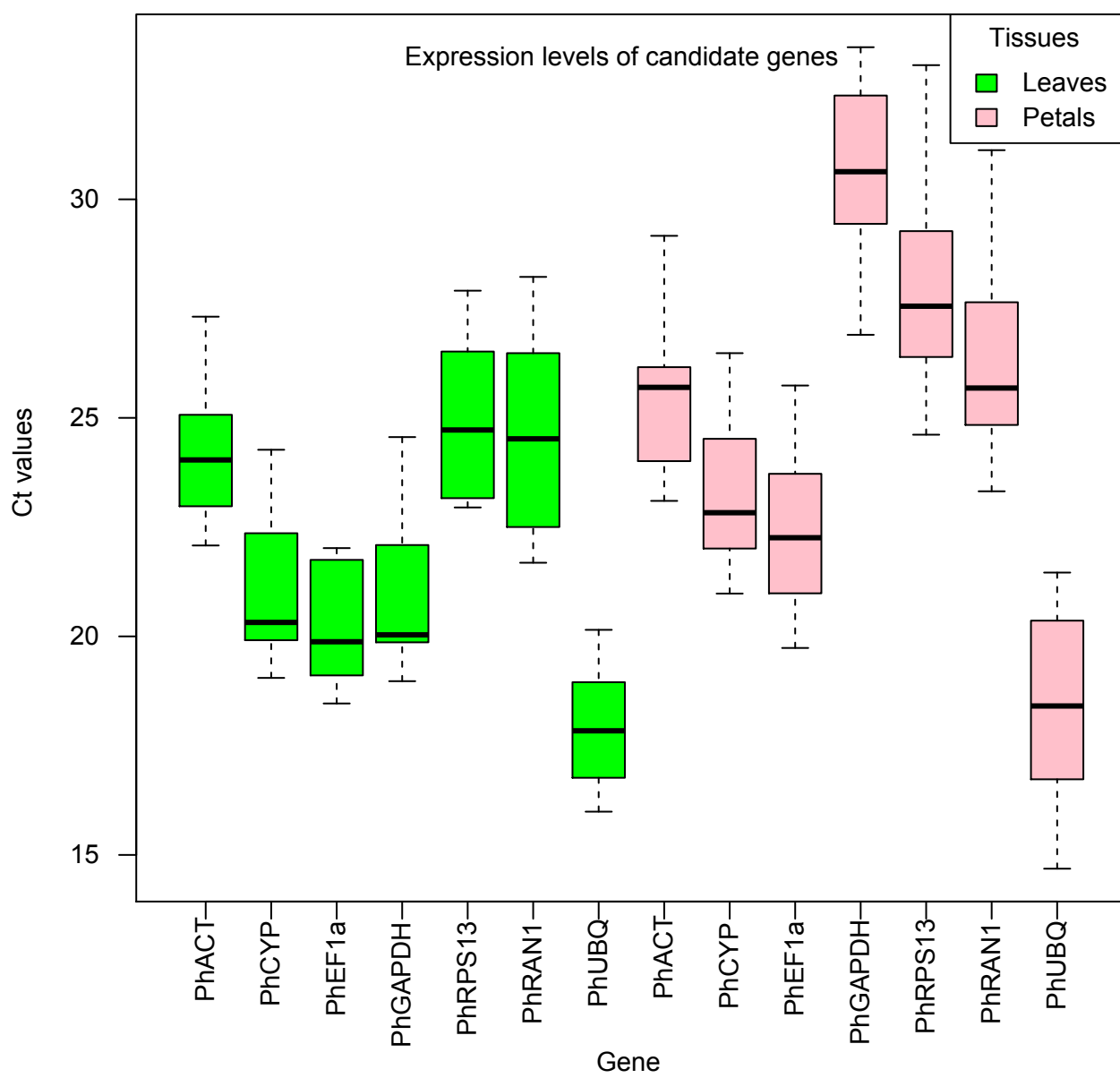

Supplement: Supplementary file 1 [file cells-08-00343-s001.zip › Supplemental Figure S1 Boxplot reference genes.pdf]

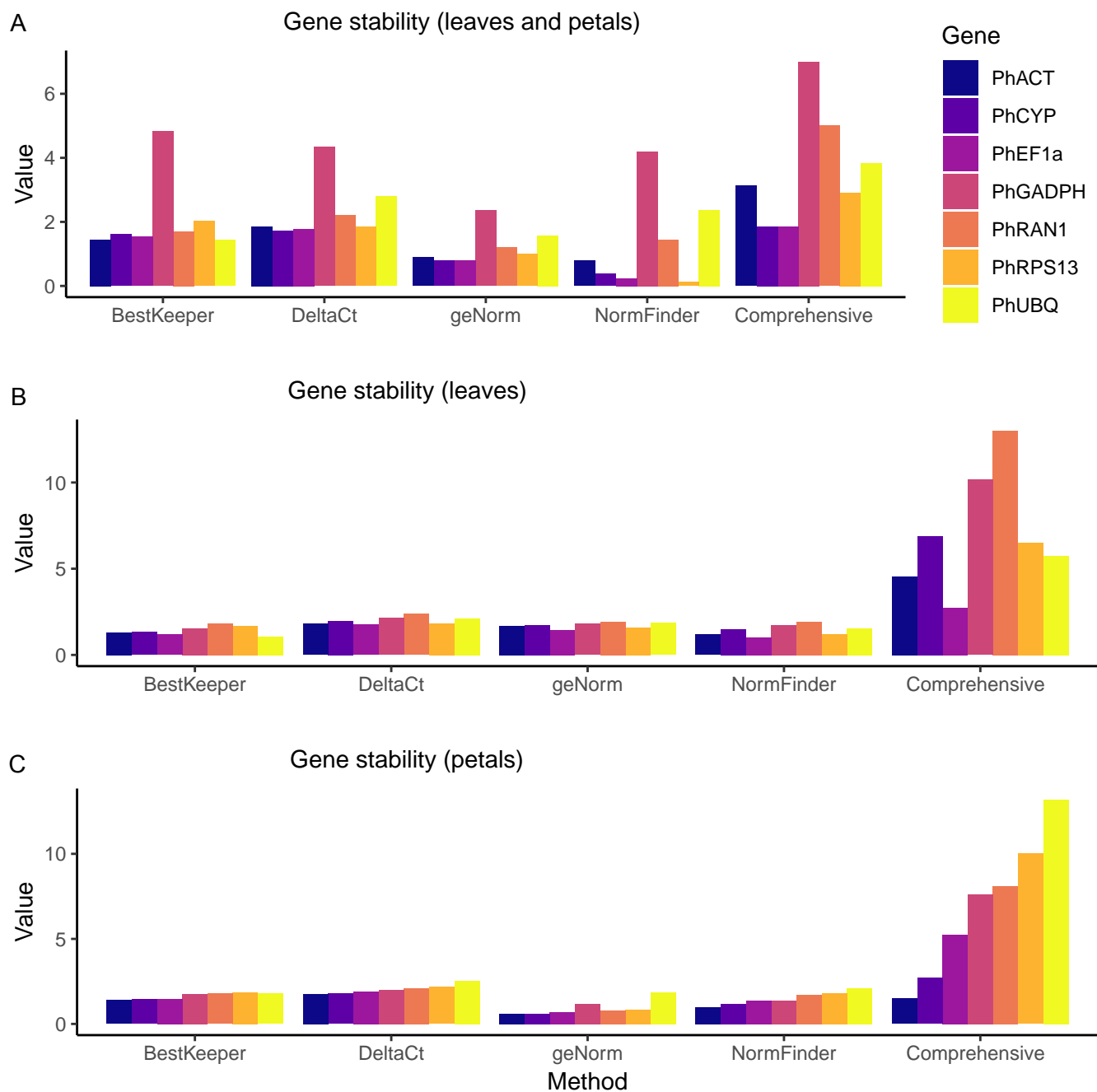

Supplement: Supplementary file 1 [file cells-08-00343-s001.zip › Supplemental Figure S2 Methods ref genes.pdf]

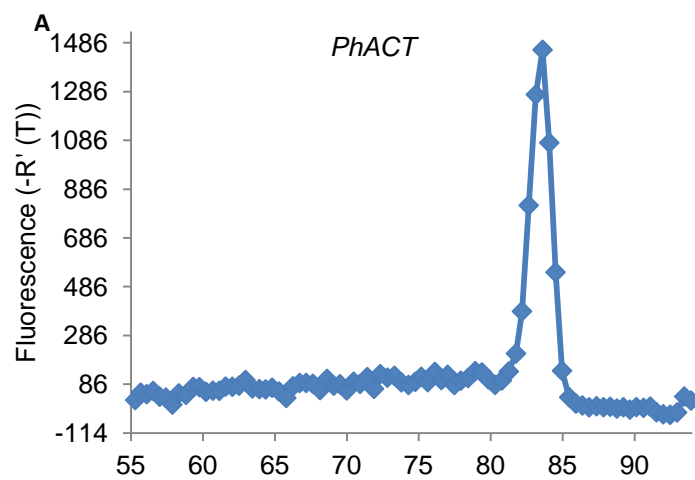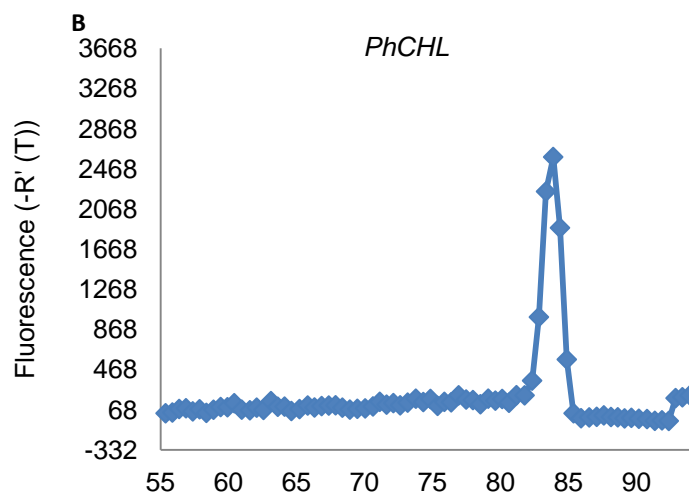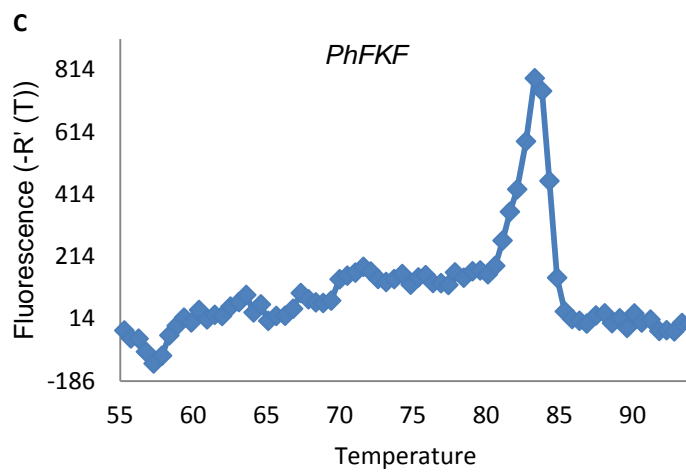

Supplement: Supplementary file 1 [file cells-08-00343-s001.zip › Supplemental Figure S3 qPCR Melting.pdf]

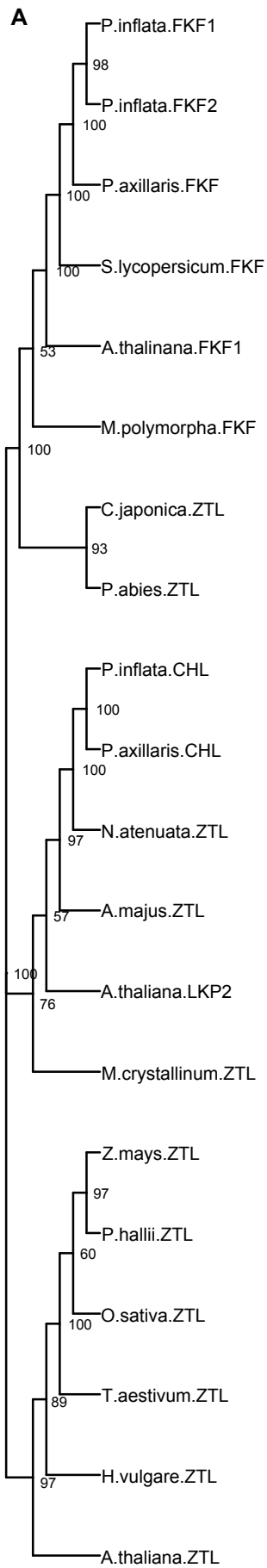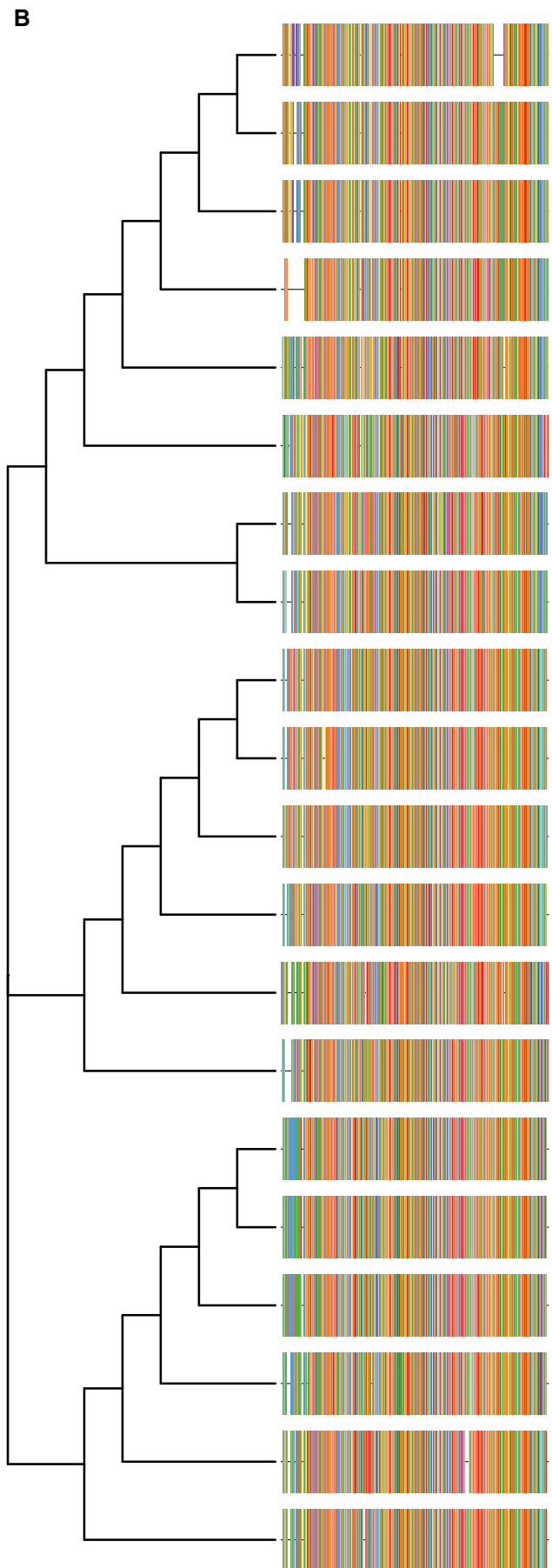

Supplement: Supplementary file 1 [file cells-08-00343-s001.zip › Supplemental Figure S4 Phylogenetic tree.pdf]

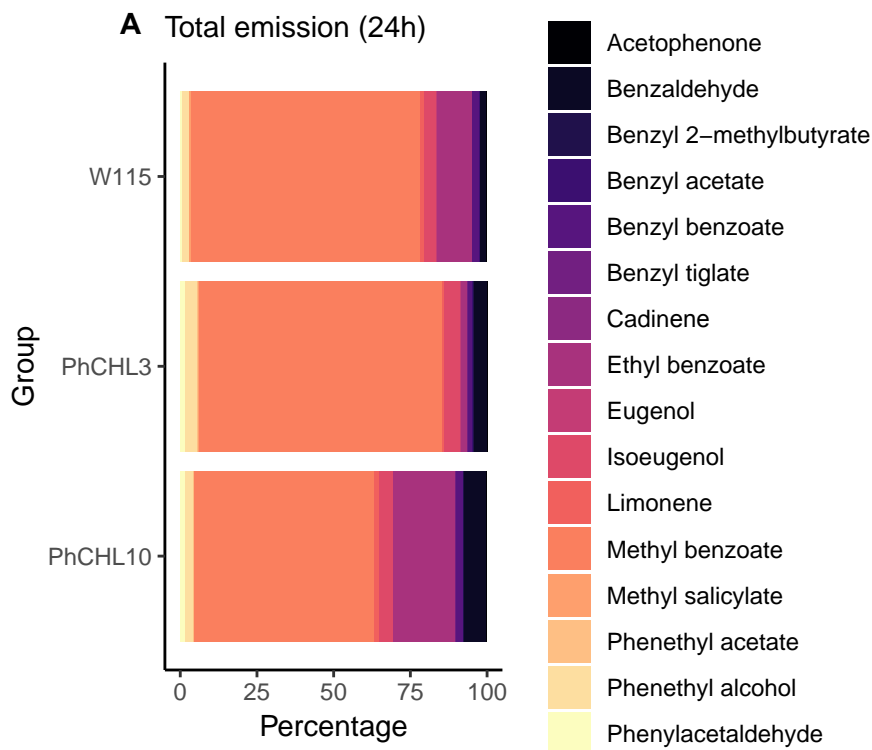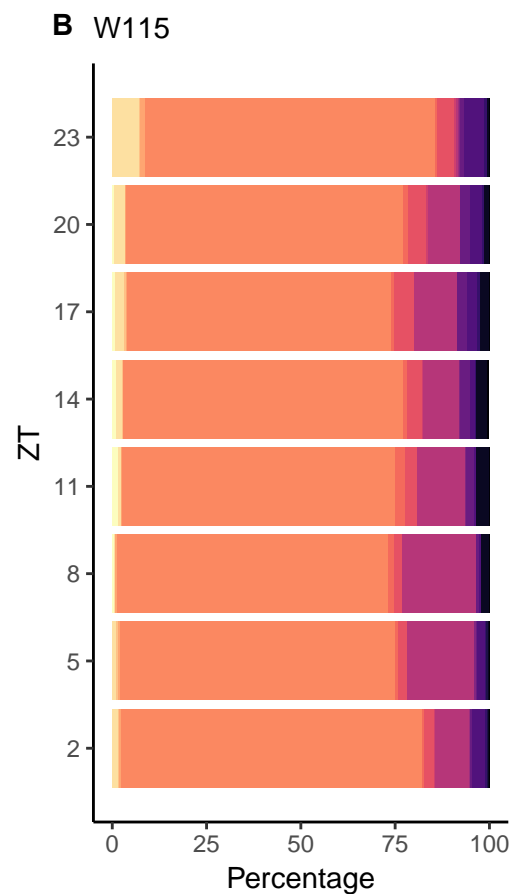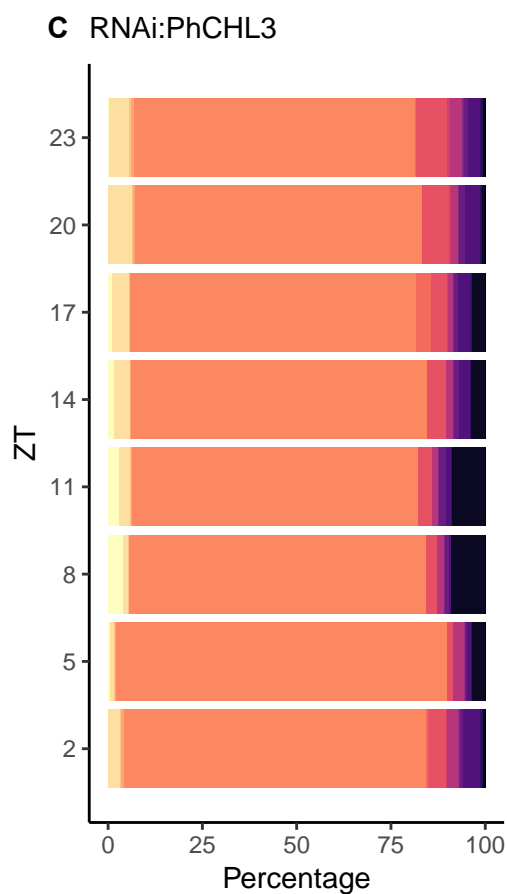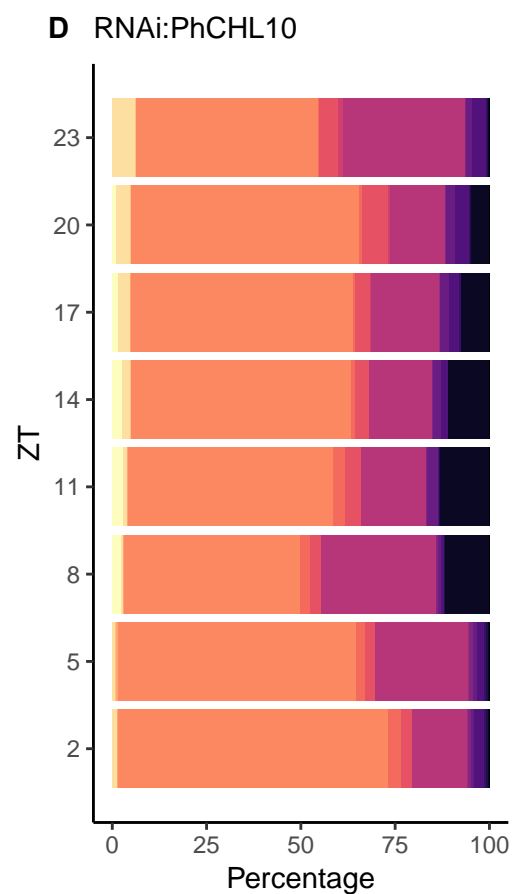

Supplement: Supplementary file 1 [file cells-08-00343-s001.zip › Supplemental Figure S5 Scent profile including methyl benzoate.pdf]
